# Validity of Using Japanese Administrative Data to Identify Inpatients With Acute Pulmonary Embolism: Referencing the COMMAND VTE Registry

**DOI:** 10.2188/jea.JE20220360

**Published:** 2024-04-05

**Authors:** Aki Kuwauchi, Satomi Yoshida, Chikashi Takeda, Yugo Yamashita, Takeshi Kimura, Masato Takeuchi, Koji Kawakami

**Affiliations:** 1 Department of Pharmacoepidemiology, Graduate School of Medicine and Public Health, Kyoto University, Kyoto, Japan; 2 Department of Anesthesia, Kyoto University Hospital, Kyoto, Japan; 3 Department of Cardiovascular Medicine, Graduate School of Medicine, Kyoto University, Kyoto, Japan

**Keywords:** validation, sensitivity, pulmonary embolism, claims data, administrative data

## Abstract

**Background:**

Acute pulmonary embolism (PE) is a life-threatening in-hospital complication. Recently, several studies have reported the clinical characteristics of PE among Japanese patients using the diagnostic procedure combination (DPC)/per diem payment system database. However, the validity of PE identification algorithms for Japanese administrative data is not yet clear. The purpose of this study was to evaluate the validity of using DPC data to identify acute PE inpatients.

**Methods:**

The reference standard was symptomatic/asymptomatic PE patients included in the COntemporary ManageMent AND outcomes in patients with Venous ThromboEmbolism (COMMAND VTE) registry, which is a cohort study of acute symptomatic venous thromboembolism (VTE) patients in Japan. The validation cohort included all patients discharged from the six hospitals included in both the registry and DPC database. The identification algorithms comprised diagnosis, anticoagulation therapy, thrombolysis therapy, and inferior vena cava filter placement. Each algorithm’s sensitivity, specificity, positive predictive value (PPV), and negative predictive value (NPV) were estimated.

**Results:**

A total of 43.4% of the validation cohort was female, with a mean age of 67.3 years. The diagnosis-based algorithm showed a sensitivity of 90.2% (222/246; 95% confidence interval [CI], 85.8–93.6%), a specificity of 99.8% (228,485/229,027; 95% CI, 99.7–99.8%), a PPV of 29.1% (222/764; 95% CI, 25.9–32.4%) and an NPV of 99.9% (228,485/229,509; 95% CI, 99.9–99.9%) for identifying symptomatic/asymptomatic PE. Additionally, 94.6% (159/168; 95% CI, 90.1–97.5%) of symptomatic PE patients were identified using the diagnosis-based algorithm.

**Conclusion:**

The diagnosis-based algorithm may be a relatively sensitive method for identifying acute PE inpatients in the Japanese DPC database.

## INTRODUCTION

Recently, real-world data, including data from electronic health records, medical claims, and other sources, have played an increasing role in observational studies.^1^ In many studies that use real-world data, the operational definitions of study elements (eg, inclusion and exclusion criteria, exposures, outcomes, key covariates) are derived from code-based algorithms using structured data elements or the extraction of relevant information from unstructured data, such as physician notes. However, because operational algorithms are usually imperfect, there is concern that the misclassification of study elements might impact the measure of associations and the interpretation of results.^2^

The diagnostic procedure combination/per diem payment (DPC/PDPS) system is a case mix-based inclusive fee schedule for inpatient care that was launched in 2002 by the Ministry of Health, Labour and Welfare (MHLW) in Japan.^3^ The DPC/PDPS system covered approximately 55% of acute general care beds nationwide in 2014.^4^ Hospitals participating in the DPC/PDPS system are obliged to submit “DPC data” to the MHLW, including discharge abstract data and claims information (regardless of reimbursement) in addition to reimbursement claims. Discharge abstract data (referred to as “Format 1”), which are created for each patient per hospitalization, are easy to analyze due to their well-organized structure and can also be utilized for research purposes. Therefore, Japanese DPC data support epidemiological studies or ongoing surveys of low-prevalence diseases, such as venous thromboembolism (VTE), although the data are limited to the in-hospital setting.

VTE, which includes pulmonary embolism (PE) and deep vein thrombosis (DVT), is the third most common acute cardiovascular condition in Western countries.^5^^–^^7^ A series of questionnaire-based reports showed that PE occurs relatively less frequently in Japan than in Western countries,^8^^–^^11^ but its incidence increased during the 1990s and 2000s.^12^ In the late 2000s, several clinical studies based on administrative data were conducted. Kunisawa et al reported that the incidence of postoperative PE was 0.05% using a diagnosis code-based algorithm for discharge abstract data from a DPC database.^13^ Additionally, Nagase et al described thromboprophylaxis and the prevalence of PE after lower extremity surgery using another DPC/claims database.^14^ However, to the best of our knowledge, the validity of PE patient identification using Japanese administrative data has not yet been well addressed. The purpose of this study was to evaluate the validity of PE patient identification using DPC data.

## METHODS

### Reference standard and validation design

Due to the low prevalence of PE, we used existing data from the COntemporary ManageMent AND outcomes in patients with Venous ThromboEmbolism (COMMAND VTE) registry^15^ as a reference standard. The design of the registry has been reported in detail elsewhere.^15^ Briefly, the registry was a physician-initiated, retrospective cohort study of consecutive patients with acute symptomatic VTE objectively confirmed by imaging examination or autopsy in 29 centers in Japan between January 2010 and August 2014. Patients were extracted from hospital databases of imaging test results (contrast-enhanced computed tomography, ultrasound, ventilation-perfusion lung scintigraphy, pulmonary angiography, or contrast venography), and diagnostic information was used as an adjunct. As a result, data from 19,634 patients suspected of VTE were extracted from hospital databases. Thereafter, the medical charts were manually reviewed, and 3,027 VTE patients were enrolled in the registry. Figure 1A shows a schematic diagram of the registry cohort, which included symptomatic PE patients with/without DVT and asymptomatic PE patients with symptomatic DVT but did not include asymptomatic PE patients with asymptomatic DVT.

**Figure 1A.**
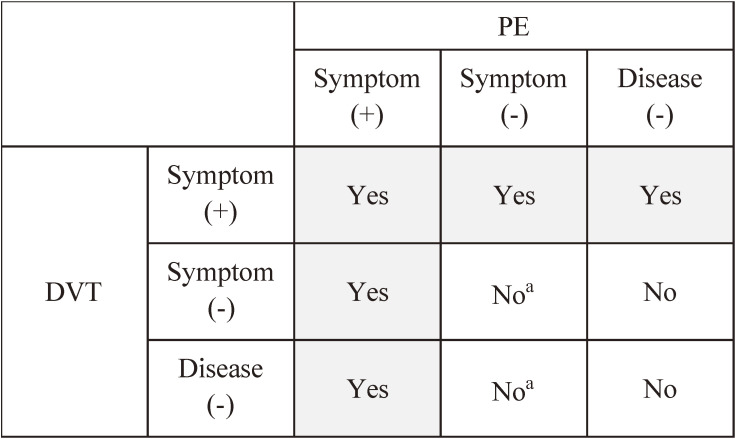
Schematic diagram of the cohort of the COMMAND VTE Registry. DVT; deep vein thrombosis, PE; pulmonary embolism, VTE; venous thromboembolism “YES” indicates the category that met the inclusion criteria for the COMMAND VTE Registry. ^a^: These categories may be one of the causes of false positives in validation because diagnosis codes in DPC data did not distinguish the existence or nonexistence of symptoms.

The flowchart of the COMMAND VTE Registry and the current study is shown in Figure 1B. The study period was from January 2010 to August 2014. The validation cohort (DPC data) included all patients discharged from the six hospitals participating in both the registry and the DPC database. Among the registry data, we selected symptomatic VTE patients from the six hospitals, and the following patients were excluded: those who were treated on an outpatient basis only, those who were diagnosed outside of the research period, and those who were discharged outside of the research period. After DPC data and registry data were linked, the reference standards (true-positive symptomatic/asymptomatic PE patients) were defined, excluding those who were not linked with DPC data and those who were diagnosed with DVT but not diagnosed with PE. Among the reference standards, the AT and TT subgroups were defined as those receiving anticoagulation therapy in the acute phase and those receiving thrombolysis therapy, respectively. Additionally, the AT+IVCf subgroup and the TT+IVCf subgroup were defined as those with IVC filter placement in addition to the corresponding therapy.

**Figure 1B.**
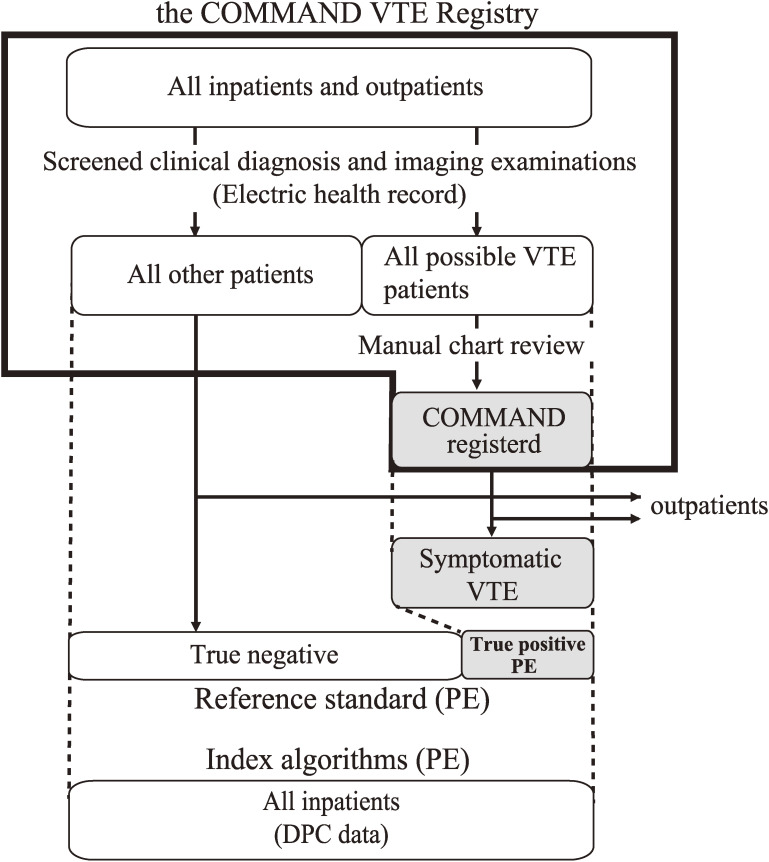
Flowchart of the COMMAND VTE Registry and the current study. PE; pulmonary embolism, VTE; venous thromboembolism

### Source of DPC data

The DPC data source was the Real World Data (RWD) database: this database is maintained by the Health, Clinic, and Education Information Evaluation Institute (HCEI; Kyoto, Japan) with support from the Real World Data Co., Ltd. (Kyoto, Japan).^16^ This database contains the records of ∼20 million patients from ∼160 medical institutions across Japan as of 2020. The stored information includes DPC data from inpatients, administrative claims data, and laboratory results from both outpatient and inpatient services. The data are automatically extracted from electronic medical records at each medical institution. Patient records are maintained by allocating unique identifiers (IDs) for each individual, which are valid within the same institution.

The DPC data consist of discharge abstract data (Format 1 file) and claims information (EF file). Format 1 files include the following data: patient demographics; diagnoses, comorbidities at admission, and complications after admission recorded by using International Classification of Diseases, Tenth Revision (ICD-10) codes and text data in Japanese; selected clinical information; admission and discharge statuses; surgeries and procedures with the original Japanese codes (K codes); and special reimbursements. EF files include the following data (regardless of reimbursement): drug administration data, medical device use data, and service data.

### Identifying PE patients in DPC data

Diagnosis information was derived from Format 1, and medication and procedure information was derived from the EF files. Five algorithms were examined in the current study: 1) diagnosis (D); 2) diagnosis and anticoagulation therapy (D+AT); 3) diagnosis, anticoagulation therapy and inferior vena cava filter placement (D+AT+IVCf); 4) diagnosis and thrombolytic therapy (D+TT); and 5) diagnosis, thrombolytic therapy and IVCf (D+TT+IVCf). eTable 1 presents the algorithm elements and the definitions. The diagnosis algorithm was developed to represent acute PE caused by thrombi, fat, and air.

### Data linkage

We were provided with COMMAND VTE Registry data and DPC data, with research IDs specific to the registry and other research IDs specific to the RWD database, respectively. Therefore, we merged the IDs from the two databases in the following two steps: 1) the correspondence table relating the hash value (generated from the hospital-specific patient ID) and the registry research ID was generated for each of the six institutions and provided to RWD Co; and 2) the correspondence table relating the registry research ID and the RWD research ID was generated at RWD Co. and provided to us. The registry data and DPC data were linked at the episode/admission level deterministically using the patient ID and the timing of the PE event. According to the timing of the event, it was considered a match if the diagnosis date in the registry existed from 6 days before the date of admission to 1 day after the date of discharge in DPC data. To assess the quality of the linkage, the following analyses were performed: 1) the linkage proportion was calculated, 2) the agreement ratio of sex and year of birth was reported, 3) the characteristics of the episodes among those whose sex or year of birth disagreed were compared, and 4) the characteristics of symptomatic VTE patients and the reference standard were described.^17^

### Main analysis and stratified analyses

We used descriptive statistics to describe the validation cohort, symptomatic VTE patients, and true-positive PE episodes. The prevalence of acute PE was defined as the proportion of true-positive PE episodes in the validation cohort.

We estimated the sensitivity, specificity, positive predictive value (PPV), and negative predictive value (NPV) with 95% confidence intervals (CIs) for binomial distributions using the exact method. We then stratified these estimates by age, sex, and setting (medical or surgical admission) to assess whether these factors significantly affected the study estimates. Surgical admissions were defined as those whose Format 1 was recorded with K codes excluding transfusion codes, pulmonary thromboendarterectomy codes (K codes: K92x, K592, K593), and any K codes under local anesthesia. For stratified analyses, the sensitivity of algorithm (D) was estimated by the following characteristics of PE episodes based on the registry data: 1) symptomatic or asymptomatic, 2) hospital-acquired or community-acquired, 3) severity classification, and 4) risk of recurrent VTE.

### Subgroup analyses and other analyses

Subgroup analyses were performed to evaluate the performance of the algorithm in identifying PE patients receiving AT, those receiving AT+IVCf, those receiving TT, and those receiving TT+IVCf. Only those who received the corresponding therapy were defined as subgroup references.

The following analyses were performed on true-positive symptomatic/asymptomatic PE episodes: 1) the position of PE diagnosis in Format 1 was described; and 2) comparisons of the treatment between DPC data and registry data were described using two-by-two tables and simple kappa statistics.

All data handling and statistical analyses were performed using SAS version 9.4 (SAS Institute Inc., Cary, NC, USA). This study was approved by the Kyoto University Graduate School and the Faculty of Medicine Ethics Committee (Kyoto, Japan, R1484). The requirement for written informed consent was waived because of the retrospective design using previously anonymized patient records. The information about this study was publicly disclosed via a webpage of Kyoto University Hospital, including an opportunity to opt out of participation.^18^

## RESULTS

### Reference standard

**Figure 2.**
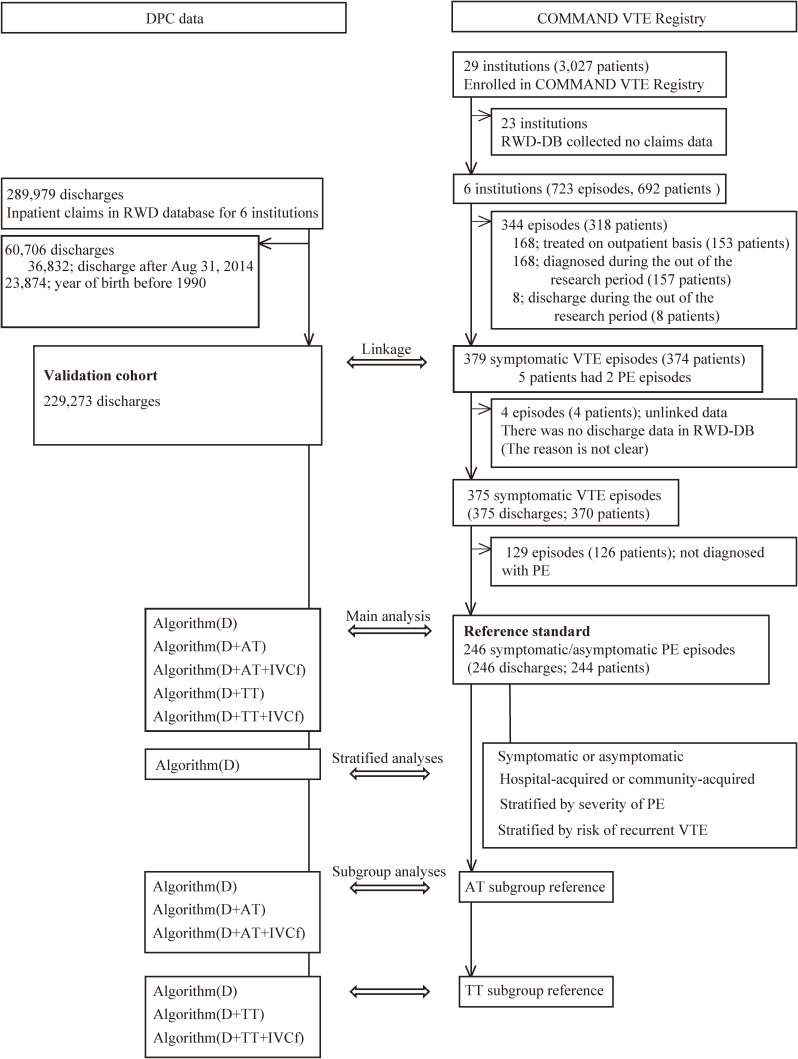
Study flow diagram. AT; anticoagulation therapy, D; diagnosis, IVCf; inferior vena cava filter placement, PE; pulmonary embolism, RWD-DB; Real World Data database, TT; thrombolysis therapy, VTE; venous thromboembolism Among the reference standards, the AT (+IVCf) and TT (+IVCf) subgroups were defined as those receiving anticoagulation therapy in the acute phase and those receiving thrombolysis therapy, respectively. The AT+IVCf and TT+IVCf subgroups were defined as those with IVC filter placement in addition to the corresponding therapy. Five algorithms were examined in the current study: 1) algorithm (D); 2) algorithm (D+AT); 3) algorithm (D+AT+IVCf); 4) algorithm (D+TT); and 5) algorithm (D+TT+IVCf).

The flowchart of the current study is shown in Figure 2. Of the 3,027 patients enrolled in the COMMAND VTE Registry, 692 patients (from six hospitals) were included. After we excluded patients who met the exclusion criteria, 375 of 379 (98.9%) symptomatic VTE episodes/discharges were successfully linked with DPC data. The agreement of both sex and year of birth was 98.7% (370/375). Five discharges with disagreement in sex or age were considered input errors in the registry or DPC data because the characteristics of the episodes/discharges were similar (data not shown). The characteristics of the symptomatic VTE patients were similar to those of the entire registry (shown in eTable 2).^15^

Among the symptomatic VTE episodes, 246 episodes/discharges were identified as the reference standard. Table 1 shows the prevalence of acute PE in each hospital. The combined average PE prevalence for the six hospitals was 7.3 and 10.7 per 10,000 discharges for symptomatic PE and asymptomatic PE, respectively. The prevalence of PE did not include cases of asymptomatic PE with asymptomatic DVT, which were not eligible for the registry. The characteristics of the true-positive PE patients (reference standard) are described in eTable 3. The proportions of symptomatic PE patients (with or without DVT) were 168/246 (68.3%) and 148/223 (66.4%) among the reference standard and AT subgroup reference, respectively. Among the TT subgroup reference, 44/49 (89.8%) had symptomatic PE.

**Table 1.** The prevalence of true-positive PE in each hospital

Hp ID	Number of discharges	Symptomatic PE		Symptomatic/asymptomatic PE	
		Number of cases	(Prevalence^a^)	Number of cases	(Prevalence^a^)
1	14,046	16	(11.4)	19	(13.5)
2	107,232	90	(8.4)	144	(13.4)
3	16,835	28	(16.6)	35	(20.8)
4	1,514	0	—	0	—
5	27,585	2	(0.7)	4	(1.5)
6	62,061	32	(5.2)	44	(7.1)
total	229,273	168	(7.3)	246	(10.7)

Hp, hospital; PE, pulmonary embolism.

^a^The prevalence of true-positive cases of PE per 10,000 discharges.

### DPC data (validation cohort)

Of the 289,979 cumulative discharges included, 60,706 were excluded following the exclusion criteria. The characteristics of the validation cohort (229,273 discharges) are shown in eTable 4. The mean age was 67.3 (standard deviation, 15.9) years, and 43.4% of the cohort was female.

### Main results

The sensitivity, specificity, PPV, NPV, and prevalence of each algorithm are shown in Table 2. The algorithm (D) showed a sensitivity of 90.2% (222/246; 95% CI, 85.8–93.6%), a specificity of 99.8% (228,485/229,027; 95% CI, 99.7–99.8%), a PPV of 29.1% (222/764; 95% CI, 25.9–32.4%) and an NPV of 99.9% (228,485/229,509; 95% CI, 99.9–99.9%). The estimates were fairly consistent across age, sex, and medical/surgical setting groups.

**Table 2.** The sensitivity, specificity, positive predictive value, and negative predictive value of each algorithm referencing the symptomatic/asymptomatic PE patients in the COMMAND VTE registry

	Sensitivity			Specificity			Prevalence^a^	
	%	(95% CI)	Number	%	(95% CI)	Number		(Number)
Algorithm (D)	90.2	(85.8–93.6)	222/246	99.8	(99.7–99.8)	228,485/229,027	10.7	(246/229,273)
Male	90.6	(82.9–95.6)	87/96	99.8	(99.8–99.8)	129,508/129,750	7.4	(96/129,846)
Female	90.0	(84.0–94.3)	135/150	99.7	(99.7–99.7)	98,977/99,277	15.1	(150/99,427)
Years of age <65	87.4	(79.0–93.3)	83/95	99.8	(99.8–99.9)	80,867/80,993	11.7	(95/81,088)
Years of age ≥65	92.1	(86.5–95.8)	139/151	99.7	(99.7–99.8)	147,618/148,034	10.2	(151/148,185)
Surgical admission	84.8	(68.1–94.9)	28/33	99.9	(99.8–99.9)	55,892/55,975	5.9	(33/56,008)
Medical admission	91.1	(86.4–94.5)	194/213	99.7	(99.7–99.8)	172,560/173,019	12.3	(213/173,232)
Algorithm (D + AT)	86.1	(81.2–90.2)	212/246	99.8	(99.8–99.8)	228,679/229,027	10.7	(246/229,273)
Algorithm (D + AT + IVCf)	30.1	(24.4–36.2)	74/246	99.9	(99.9–99.9)	228,970/229,027	10.7	(246/229,273)
Algorithm (D + TT)	17.9	(13.3–23.3)	44/246	99.9	(99.9–99.9)	229,004/229,027	10.7	(246/229,273)
Algorithm (D + TT + IVCf)	8.1	(5.0–12.3)	20/246	99.9	(99.9–99.9)	229,021/229,027	10.7	(246/229,273)



	Positive Predictive Value			Negative Predictive Value		
	%	(95% CI)	Number	%	(95% CI)	Number
Algorithm (D)	29.1	(25.9–32.4)	222/764	99.9	(99.9–99.9)	228,485/228,509
Male	26.4	(21.8–31.6)	87/329	99.9	(99.9–99.9)	228,485/129,517
Female	31.0	(26.7–35.6)	135/435	99.9	(99.9–99.9)	228,691/98,992
Years of age <65	39.7	(33.0–46.7)	83/209	99.9	(99.9–99.9)	229,199/80,879
Years of age ≥65	25.0	(21.5–28.9)	139/555	99.9	(99.9–99.9)	147,618/147,630
Surgical admission	25.2	(17.5–34.4)	28/111	99.9	(99.9–99.9)	55,892/55,975
Medical admission	29.7	(26.2–33.4)	194/653	99.9	(99.9–99.9)	172,560/172,579
Algorithm (D + AT)	37.9	(33.8–42.0)	212/560	99.9	(99.9–99.9)	228,679/228,713
Algorithm (D + AT + IVCf)	56.5	(47.6–65.1)	74/131	99.9	(99.9–99.9)	228,970/229,142
Algorithm (D + TT)	65.7	(53.1–76.8)	44/67	99.9	(99.9–99.9)	229,004/229,206
Algorithm (D + TT + IVCf)	76.9	(56.4–91.0)	20/26	99.9	(99.9–99.9)	229,021/229,247

AT, anticoagulation therapy; CI, confidence interval; IVCf, inferior vena cava filter placement; PE, pulmonary embolism; TT, thrombolysis therapy; VTE, venous thromboembolism.

AT was defined as the administration of unfractionated heparin or fondaparinux and warfarin during hospitalization. TT was defined as the administration of monteplase or urokinase during hospitalization.

^a^The prevalence of true-positive cases of symptomatic/asymptomatic PE per 10,000 discharges.

The algorithm (D+AT) resulted in a slightly higher PPV of 37.9% (212/560; 95% CI, 33.8–42.0%) compared to that of the algorithm (D). The algorithm (D+TT) showed a much higher PPV of 65.7% (44/67; 95% CI, 53.1–76.8%). The sensitivities of algorithm (D+AT+IVCf) and algorithm (D+TT+IVCf) were significantly lower; on the other hand, the PPVs tended to be higher compared to those of algorithm (D+AT) and algorithm (D+TT), respectively.

Table 3 shows the results of the stratified analyses. A total of 94.6% (159/168; 95% CI, 90.1–97.5%) of symptomatic PE episodes were identified using the algorithm (D). Among the nine false-negative symptomatic PE episodes, five had DVT diagnoses or codes for IVC filter placements, one had a suspected PE diagnosis (this patient presented with cardiac arrest and died on the third hospital day), one had a chronic PE diagnosis, and the other two had no diagnoses or codes relating to VTE.

**Table 3.** The sensitivity of diagnosis-based algorithm by patient characteristic based on the registry (stratified analyses)

Strata (Reference)	Algorithm	Sensitivity		
		%	(95% CI)	Number
Symptoms				
Symptomatic PE	Algorithm (D)	94.6	(90.1–97.5)	159/168
Asymptomatic PE	Algorithm (D)	80.8	(70.3–88.8)	63/78
Onset				
Community-acquired PE	Algorithm (D)	93.0	(88.5–96.1)	185/199
Hospital-acquired PE (after surgery)	Algorithm (D)	75.0	(55.1–89.3)	21/28
Hospital-acquired PE (others)	Algorithm (D)	84.2	(60.4–96.6)	16/19
Severity classification				
Cardiac arrest or collapse	Algorithm (D)	90.9	(58.7–99.8)	10/11
Massive	Algorithm (D)	92.9	(66.1–99.8)	13/14
Submassive	Algorithm (D)	94.2	(87.8–97.8)	97/103
Nonmassive	Algorithm (D)	86.4	(78.9–92.0)	102/118
Risk of recurrent VTE				
Unprovoked group	Algorithm (D)	96.0	(91.0–98.7)	121/126
Transient risk group	Algorithm (D)	84.8	(75.0–91.9)	67/79
Active cancer group	Algorithm (D)	82.9	(68.0–92.8)	34/41

CI, confidence interval; PE, pulmonary embolism.

The subgroup validation results are shown in Table 4. Algorithm (D) showed a sensitivity of 91.5% (204/223; 95% CI, 87.0–94.8%), 92.6% (63/68; 95% CI, 83.7–97.6%), 95.9% (47/49; 95% CI, 86.0–99.5%), and 95.5% (21/22; 95% CI, 77.2–99.9%) for the AT, AT+IVCf, TT, and TT+IVCf subgroups, respectively. The sensitivities of algorithm (D+AT+IVCf) and algorithm (D+TT+IVCf) were significantly lower; on the other hand, the PPVs tended to be higher compared to those of algorithm (D+AT) and algorithm (D+TT).

**Table 4.** The algorithm validation results for PE patient subgroups

Subgroup (reference)	Algorithm	Sensitivity			Specificity			Prevalence^a^	
		%	(95% CI)	Number	%	(95% CI)	Number		(Number)
AT	Algorithm (D)	91.5	(87.0–94.8)	204/223	99.8	(99.7–99.8)	228,490/229,050	9.7	(223/229,273)
	Algorithm (D + AT)	90.6	(86.0–94.1)	202/223	99.8	(99.8–99.9)	228,692/229,050	9.7	(223/229,273)
	Algorithm (D + AT + IVCf)	30.5	(24.5–37.0)	68/223	99.9	(99.9–99.9)	228,987/229,050	9.7	(223/229,273)

AT + IVCf	Algorithm (D)	92.6	(83.7–97.6)	63/68	99.7	(99.7–99.7)	228,504/229,205	3.0	(68/229,273)
	Algorithm (D + AT)	92.6	(83.7–97.6)	63/68	99.8	(99.8–99.8)	228,708/229,205	3.0	(68/229,273)
	Algorithm (D + AT + IVCf)	91.2	(81.8–96.7)	62/68	99.9	(99.9–99.9)	229,136/229,205	3.0	(68/229,273)

TT	Algorithm (D)	95.9	(86.0–99.5)	47/49	99.7	(99.7–99.7)	228,507/229,224	2.1	(49/229,273)
	Algorithm (D + TT)	85.7	(72.8–94.1)	42/49	99.9	(99.9–99.9)	229,199/229,224	2.1	(49/229,273)
	Algorithm (D + TT + IVCf)	40.8	(27.0–55.8)	20/49	99.9	(99.9–99.9)	229,218/229,224	2.1	(49/229,273)

TT + IVCf	Algorithm (D)	95.5	(77.2–99.9)	21/22	99.7	(99.7–99.7)	228,508/229,251	1.0	(22/229,273)
	Algorithm (D + TT)	90.9	(70.8–98.9)	20/22	99.9	(99.9–99.9)	229,204/229,251	1.0	(22/229,273)
	Algorithm (D + TT + IVCf)	86.4	(65.1–97.1)	19/22	99.9	(99.9–99.9)	229,244/229,251	1.0	(22/229,273)



Subgroup (reference)	Algorithm	Positive Predictive Value			Negative Predictive Value		
		%	(95% CI)	Number	%	(95% CI)	Number
AT	Algorithm (D)	26.7	(23.6–30.0)	204/764	99.9	(99.9–99.9)	228,490/228,509
	Algorithm (D + AT)	36.1	(32.1–40.2)	202/560	99.9	(99.9–99.9)	228,692/228,713
	Algorithm (D + AT + IVCf)	51.9	(43.0–60.7)	68/131	99.9	(99.9–99.9)	228,987/229,142

AT + IVCf	Algorithm (D)	8.2	(6.4–10.4)	63/764	99.9	(99.9–99.9)	228,504/228,509
	Algorithm (D + AT)	11.3	(8.8–14.2)	63/560	99.9	(99.9–99.9)	228,708/228,713
	Algorithm (D + AT + IVCf)	47.3	(38.5–56.2)	62/131	99.9	(99.9–99.9	229,136/229,142

TT	Algorithm (D)	6.2	(4.6–8.1)	47/764	99.9	(99.9–99.9)	228,507/228,509
	Algorithm (D + TT)	62.7	(50.0–74.2)	42/67	99.9	(99.9–99.9)	229,199/229,206
	Algorithm (D + TT + IVCf)	76.9	(56.4–91.0)	20/26	99.9	(99.9–99.9)	229,218/229,247

TT + IVCf	Algorithm (D)	2.7	(1.7–4.2)	21/764	99.9	(99.9–99.9)	228,508/228,509
	Algorithm (D + TT)	29.9	(19.3–42.3)	20/67	99.9	(99.9–99.9)	229,204/229,206
	Algorithm (D + TT + IVCf)	73.1	(52.2–88.4)	19/26	99.9	(99.9–99.9)	229,244/229,247

AT, anticoagulation therapy; CI, confidence interval; IVCf, inferior vena cava filter placement; TT, thrombolysis therapy.

The AT subgroup comprised symptomatic/asymptomatic PE patients receiving AT.

The AT + IVCf subgroup was consisted by PE patients receiving AT and IVCf.

AT was defined as the administration of unfractionated heparin or fondaparinux and warfarin during hospitalization.

The TT subgroup comprised symptomatic/asymptomatic PE patients receiving TT.

The TT + IVCf subgroup comprised PE patients receiving TT and IVCf.

TT was defined as the administration of monteplase or urokinase during hospitalization.

^a^The prevalence of true-positive cases per 10,000 discharges.

The position of PE diagnoses in Format 1 among true-positive PE patients is described in Table 5. In 10 of 28 patients with hospital-acquired PE who were diagnosed within 2 months of surgery, PE diagnoses were due to comorbidities at the time of admission.

**Table 5.** The position of PE diagnosis in discharge abstract data (Format 1 file) among true-positive symptomatic/asymptomatic PE patients (246 patients)

PE onset	Main condition		Trigger-for- hospitalization condition		Greatest-resource condition		Second greatest- resource condition		Comorbidities at the time of admission		Conditions occurring during the hospitalization		All	
	*n*	(%)	*n*	(%)	*n*	(%)	*n*	(%)	*n*	(%)	*n*	(%)	*n*	(%)
Out of the hospital	158	(79.4)	155	(77.9)	157	(78.9)	0	(0.0)	25	(12.6)	3	(1.5)	199	(100.0)
Hospital-acquired PE (after surgery)^a^	0	(0.0)	0	(0.0)	0	(0.0)	6	(21.4)	10	(35.7)	12	(42.9)	28	(100.0)
Hospital-acquired PE (others)	10	(52.6)	6	(31.6)	10	(52.6)	4	(21.1)	3	(15.8)	4	(21.1)	19	(100.0)
Overall	168	(68.3)	161	(65.5)	167	(67.9)	10	(4.1)	38	(15.5)	19	(7.7)	246	(100.0)

PE, pulmonary embolism.

^a^Hospital-acquired PE episode that was diagnosed within 2 months of surgery.

eTable 5 presents comparisons of treatment in DPC data and registry data among the true-positive PEs. The agreement ratios of “UFH or fondaparinux” and warfarin were 89.0% (216/219) and 87.3% (215/246), respectively; however, the kappa statistics were 0.15 and 0.39, respectively. The kappa statistics of thrombolysis therapy and IVC filter placement were 0.88 and 0.91, respectively.

## DISCUSSION

The diagnosis information in Format 1 in the DPC database enabled us to detect more than 90% of the acute PE inpatients in our validation cohort. The validity was fairly consistent across age, sex, and medical/surgical setting groups. The diagnosis-based algorithm also had high sensitivity in identifying subgroups of acute PE inpatients receiving anticoagulation therapy or thrombolysis therapy. This is the first report on the sensitivity of PE patient identification using Japanese administrative data.

The validity of PE patient identification using Japanese administrative data, especially sensitivity, is difficult to estimate because of the low disease prevalence.^12^ To overcome this challenge, an existing registry was used as a reference standard in our study. All discharges in DPC data were tested using the index algorithm; however, not all of them were tested using the reference standard because “all possible patients” were manually reviewed in the process of registry inclusion. This is a modified stratification design technique that is applicable for diagnostic accuracy studies in low-prevalence situations.^19^ In the current study, this technique is performed on the assumption that there is no true-positive patient other than those listed as “all possible patients”. Several studies regarding the accuracy of multiple sclerosis identification used this technique, reporting that a random sample would not have yielded enough cases due to the low prevalence.^20^^,^^21^

The PPV of algorithm (D) was lower than 30%, and adding the treatment element to the algorithm had a marginal effect on the PPV. However, our PPV estimates should be considered to be underestimated. This is because the asymptomatic PE patients, who were detected as PE patients by DPC data, decreased the PPV because they were not eligible for inclusion in the registry. To support this, the higher proportion of symptomatic patients among the TT subgroup reference resulted in a higher PPV in algorithm (D+TT) than in algorithm (D).

The addition of IVC filter placement to algorithm (D+AT) and algorithm (D+TT) tended to increase the PPVs. Moreover, the sensitivities for the AT+IVCf and TT+IVCf subgroups remained high. Therefore, these algorithms would be useful when a researcher is interested in PE patients undergoing IVC filter placement.

Differentiating between hospital-acquired and community-acquired onset is important for future clinical studies. In stratified analyses, the sensitivity of hospital-acquired PE tended to be lower than that of community-acquired PE (Table 3). However, it was not possible to distinguish between patients with and without onset during hospitalization. This is because hospital-acquired PE patients in the registry included those who developed PE once discharged from the hospital after surgery or those who developed it during treatment (eg, chemotherapy for cancer) in the outpatient department of that hospital. This can be inferred from the fact that some patients with hospital-acquired PE had the diagnosis of “trigger-for-hospitalization condition” or “comorbidities at the time of admission” (Table 5).

We compared the treatment for true-positive PE episodes between DPC data and registry data. According to treatment with thrombolysis therapy and IVC filter placement, the records revealed excellent reproducibility, which is consistent with the findings of previous studies.^22^ However, according to anticoagulation therapy, the reproducibility was poor, although the agreement ratio was high. One possible reason for this is that we defined one or more prescriptions as the treatment in our study. Further research is needed to create a more valid algorithm for anticoagulation therapy for PE patients.

In the design of epidemiological studies using databases, the use of an algorithm with high sensitivity is important when the goal is to identify all persons with certain characteristics.^23^ Our findings, which showed that the algorithms were highly sensitive, suggest that PE identification using DPC data could be a powerful screening tool for surveillance studies with confirmation via manual chart review. When designing an outcome study, the use of an algorithm with a high PPV is important in the development of an appropriate cohort, and equivalent sensitivity among groups is needed for the precise estimation of relative risks.^23^ Therefore, manual chart review after screening using DPC data enables researchers to develop a cohort without false-positive participants. In both design situations, highly sensitive algorithms are important for the generalizability of results because less sensitive algorithms may be differentially sensitive to different disease characteristics.^23^

This is the first study to evaluate the validity of PE identification algorithms in Japanese administrative data using registry data as the reference standard. Furthermore, the strength of this study is that it estimated sensitivity, which is difficult to measure due to the low frequency of PE. Therefore, our results would be helpful for future clinical studies using Japanese DPC data for acute hospital inpatients, even when only the PPV is validated. In addition, the diagnosis algorithm could be applied to research using claims data among hospitals participating in the DPC/PDPS system because claims data include the “SB records”, which are identical to diagnosis information in format 1 of DPC data.

Our study had several limitations. First, true-positive PE patients were possibly missed because of the assumption that there were no true-positive patients other than those listed as “all possible patients”. However, data from 19,634 “possible patients” were extracted through screening for imaging examination results and clinical diagnosis information. We believe that this was the best available method to determine the presence of the target condition. Therefore, the PE patients confirmed by the chart review for “all possible patients” in the registry were justified as a reference standard.

Second, the results of the current study have limited generalizability because only patients treated at six institutions were included. This is also because the institutions participating in the COMMAND VTE Registry may have better disease recording or clinical practice patterns. Ideally, a validation study should be conducted in their study population because the performance of an algorithm is dependent on various factors, such as the data source, study population, and choice of the reference standard.^2^ However, it seems infeasible for a manual chart review to measure a reference standard because the prevalence of PE in Japan is very low. Although our validation cohort was limited to patients in six institutions, the baseline characteristics of our patients were consistent with those of patients in general acute hospitals in a report by the MHLW.^24^ In addition, the prevalence and characteristics of true-positive PE patients in the current study were consistent with those of previous reports.^12^^,^^13^^,^^25^^,^^26^

Third, the PPV was underestimated because asymptomatic PE patients without VTE were not eligible for inclusion in the registry. The PPV is easy to measure even for diseases with a low prevalence because only algorithm-positive patients are included in the reference standard test (eg, manual chart review). Therefore, for each study in the future, the PPV should be evaluated using a validation cohort that replicates the target population.

Fourth, direct oral anticoagulants were not yet approved during the period of this study. Because some of the direct oral anticoagulants are used for initial therapy for PE patients except among critically ill patients,^27^ the definition of anticoagulation therapy needs to be modified for the period when they became clinically available.

In conclusion, PE diagnostic codes obtained from Japanese DPC data may provide a relatively sensitive method to identify inpatients with acute PE, especially symptomatic patients. Highly sensitive algorithms can be useful screening tools for surveillance studies. Additionally, these algorithms may extract appropriate PE cohorts with high generalizability when combined with confirmation using manual chart review. However, the PPV should be evaluated as a part of future individual research because it was underestimated in the current study.

## SUPPLEMENTARY MATERIAL

eTable 1. Algorithm elements and definitions

eTable 2. Characteristics of symptomatic VTE patients (370 patients^a^)

eTable 3. Characteristics of true positive pulmonary embolism episodes (reference standard) in the COMMAND VTE Registry (246 episodes)

eTable 4. Characteristics of the validation cohort (229,273 discharges)

eTable 5. Comparison of the treatment for true-positive pulmonary embolism patients between DPC data and registry data (246 episodes)
