# Supplementary material for: Validity of Using Japanese Administrative Data to Identify Inpatients With Acute Pulmonary Embolism: Referencing the COMMAND VTE Registry
Source: J Epidemiol. 2024 Apr 5;34(4):155–63. doi: 10.2188/jea.JE20220360 (PMC10918337; doi:10.2188/jea.JE20220360)
Supplement: Supplementary file 1 [file je-34-155-s001.pdf]

eTable 1. Algorithm elements and definitions

Algorithm element	Definition of the element	Codes
Diagnosis	Inclusion; pulmonary embolism<br><br><br>Exclusion;<br>chronic ("mansei" in Japanese) or suspicious ("utagai" in Japanese) in diagnosis field	ICD-10 code in any diagnosis field in discharge abstract data (Format 1 file)<br>I260, I269, O882, O880, O888, T790, T791
Anticoagulation therapy	Prescription during hospitalization<br>(UFH or fondaparinux) and Warfarin	Medication code in prescription data (EF file)<br>Warfarin:<br>610450012,610460002,610462024,610462025,610463227,610463228,613330001,613330002,613330003,613330004,620000730,620000731,620002332,620002472,620002473,620811502,620811503,620811507,620811510,620811511,621480504,621480506,621480507,621480604,621938101,621940901,622122601<br>UFH:<br>621825102,621824802,621824902,622458001,620812203,621825502,621825302,621824702,621825002,621933401,621826402,621825802,621825704,621826102,621826004,621825602,620812504,620006728,620006739,620006725,643330011,620006734,620002286,620002287,620003099,620003100,620003101,620004326,620004327,620006720,620006723,620006726,620006727,620006729,620006731,620006732,620006733,620006736,620006737,620006738,620006740,620006741,620006742,620007487,620007488,620007489,620007490,620007491,620007492,620008392,620008393,620008394,620008395,621825301,621825401,621825501,621825701,621825901,621825903,621826001,621826301,640450015,643330004,643330007,643330008,643330009,643330010,643330012,643330021,643330022,643330024,643330025,643330026,643330027,643330029,643330030,643330031,643330033,643330034,643330035,643330037<br>Fondaparinux: 620004874,620004875,622044501,622044601
Thrombolysis therapy[a]	Administration of tPA or uPA during hospitalization	Medication code in EF file<br>Monteplase:<br>620007270,620007271,620007272,640421045,640421046,640421047<br>Urokinase:<br>620006202,620006203,620006204,640463028,640463029,640463030,

	643950042,643950044,643950052
IVC filter placement	K code in Format 1; K620

IVC, inferior vena cava; PE, pulmonary embolism; tPA, tissue plasminogen activator; UFH, unfractionated heparin; uPA, urokinase-type plasminogen activator; VTE, venous thromboembolism.

[a] Low-molecular-weight heparin was not reimbursable for VTE patients with Japanese health insurance. Monteplase was the only approved medicine for systemic thrombolysis in acute PE patients with Japanese health insurance during the study period.

eTable 2. Characteristics of symptomatic VTE patients (370 patients[a])

Characteristics	
Age, mean (SD)	68.7 (15.0)
Female sex, n (%)	222 (60.0)
BMI, mean (SD)	23.6 (5.1)
Hypertension, n (%)	175 (47.3)
Diabetes mellitus, n (%)	42 (11.4)
Dyslipidemia, n (%)	84 (22.7)
Chronic kidney disease, n (%)	72 (19.5)
Dialysis, n (%)	2 (0.5)
Chronic lung disease, n (%)	35 (9.5)
Previous heart failure, n (%)	15 (4.1)
Previous MI, n (%)	16 (4.3)
Previous stroke, n (%)	40 (10.8)
Atrial fibrillation, n (%)	14 (3.8)
Liver cirrhosis, n (%)	4 (1.1)
Connective tissue disease, n (%)	25 (6.8)
Risk of recurrent VTE	
Unprovoked group	169 (45.7)
Transient risk group	142 (38.4)
Active cancer group	59 (15.9)

BMI, body mass index; MI, myocardial infarction; VTE, venous thromboembolism.

[a] A total of 375 VTE episodes occurred among 370 patients.

eTable 3. Characteristics of true positive pulmonary embolism episodes (reference standard) in the COMMAND VTE Registry (246 episodes)

Characteristics	
Age, mean (SD)	67.1 (14.4)
Female sex, n (%)	150 (61.0)
Active cancer, n (%)	41 (16.7)
Prolonged immobilization, n (%)	35 (14.2)
Recent surgery[a], n (%)	34 (13.8)
DVT, n (%)	200 (81.3)
Obesity[b], n (%)	23 (9.3)
Onset, n (%)	
Community-acquired	199 (80.9)
Hospital-acquired	47(19.1)
Presentation, n (%)	
Symptomatic	168 (68.3)
Asymptomatic	78 (31.7)
Severity classification, n (%)	
Cardiac arrest or collapse	11 (4.5)
Massive	14 (5.7)
Submassive	103 (41.9)
Nonmassive	118 (48.0)
Treatment, n (%)	
Unfractionated heparin in acute phase	241 (98.0)
Fondaparinux in acute phase	4 (1.6)
Anticoagulation in acute and chronic phases	223 (90.7)
Thrombolysis therapy	49 (19.9)
Outcome, n (%)	
30-day mortality	15 (6.1)

DVT, deep vein thrombosis.

[a] surgery within 2 months prior to pulmonary embolism diagnosis

[b] body mass index ≥30 kg/m^2

eTable 4. Characteristics of the validation cohort (229,273 discharges)

Characteristics	
Age, mean (SD)	67.3 (15.9)
Female sex, n (%)	99,427 (43.4)
BMI, mean (SD)	22.7 (15.0)
Smoking, mean (SD)	247 (581)
In-hospital mortality, n (%)	10,235 (4.5)
Pregnancy, n (%)	6,511 (2.8)
Admission to a psychiatric ward, n (%)	45 (0.0)
Hospitalization for chemotherapy, n (%)	2,265 (1.0)
Surgical admission, n (%)	56,041 (24.4)
Length of hospital stay, days, mean (SD)	13.8 (23.6)
Major diagnostic category, n (%)	
missing	40,206 (17.5)
01; Neurological	10,151 (5.4)
02; Ophthalmological	8,781 (4.6)
03; Otolaryngological	6,743 (3.6)
04; Respiratory system	21,728 (11.5)
05; Cardiovascular	31,630 (16.7)
06; Gastrointestinal	45,590 (24.1)
07; Musculoskeletal system	8,129 (4.3)
08; Skin and subcutaneous tissue	2,232 (1.2)
09; Breast	2,595 (1.4)
10; Endocrine, nutritional and metabolic	6,917 (3.7)
11; Genitourinary system	14,709 (7.8)
12; Pregnancy, childbirth, and the puerperium	11,318 (6.0)
13; Blood-forming organs and immune system	6,181 (3.3)
14; Neonate and congenital malformations	229 (0.1)
15; Pediatric	750 (0.4)
16; Trauma, burn, and poisoning	9,524 (5.0)
17; Mental and behavioral	50 (0.03)
18; Others	1,810 (1.0)

BMI, body mass index; VTE, venous thromboembolism.

eTable 5. Comparison of the treatment for true-positive pulmonary embolism patients between DPC data and registry data (246 episodes)

κ =0.15		Registry data: UFH or fondaparinux in the acute phase					
		No		Yes		All	
		n	(%)	n	(%)	n	(%)
DPC data	No	3	(60.0)	25	(10.4)	28	(11.4)
UFH or fondaparinux	Yes	2	(40.0)	216	(89.6)	218	(88.6)
	All	5	(100.0)	241	(100.0)	246	(100.0)

κ =0.39		Registry data: Warfarin beyond the acute phase*					
		No		Yes		All	
		n	(%)	n	(%)	n	(%)
DPC data	No	13	(56.5)	21	(9.4)	34	(13.8)
Warfarin	Yes	10	(43.5)	202	(90.6)	212	(86.2)
	All	23	(100.0)	223	(100.0)	246	(100.0)

κ =0.88		Registry data: Thrombolysis therapy					
		No		Yes		All	
		n	(%)	n	(%)	n	(%)
DPC data	No	195	(99.0)	7	(14.3)	202	(82.1)
Thrombolysis therapy	Yes	2	(1.0)	42	(85.7)	44	(17.9)
	All	197	(100.0)	49	(100.0)	246	(100.0)

κ =0.91		Registry data: IVC filter placement					
		No		Yes		All	
		n	(%)	n	(%)	n	(%)
DPC data	No	12	(100.0)	2	(2.7)	14	(16.1)
IVC filter placement	Yes	0	(0.0)	73	(97.3)	73	(83.9)
	All	12	(100.0)	75	(100.0)	87	(100.0)

DPC, diagnosis procedure combination; IVC, inferior vena cava; κ , simple kappa statistics; UFH, unfractionated heparin.
